# Integrating Kindergartener-Specific Questionnaires With Citizen Science to Improve Child Health

**DOI:** 10.3389/fpubh.2018.00236

**Published:** 2018-08-28

**Authors:** Peng Jia

**Affiliations:** ^1^Faculty of Geo-information Science and Earth Observation (ITC), University of Twente, Enschede, Netherlands; ^2^International Initiative on Spatial Lifecourse Epidemiology (ISLE), Enschede, Netherlands

**Keywords:** child health, child abuse, kindergartener, interactive questionnaire, geographic information systems, volunteered geographic information, crowdsourcing science

## Background

On November 24, 2017, the State Council of China issued an urgent notice concerning launching a nationwide inspection of the standardization of kindergarten management and education. Triggered by several recent kindergarten abuse claims, China is aiming to reduce the occurrence of such cases in an effective way. From a lifecourse epidemiological perspective, such abuse experiences in the early life can jeopardize children's behavior, physical and especially mental health in adulthood (1–5). There are a total estimation of 239 thousand kindergartens and 82 million population aged 0–4 in China by 2016 (6, 7). Therefore, such a nationwide inspection could be considered a large-scale public health intervention program. These recent abuse cases, found out accidentally by parents at home, imply that an unknown number of adolescent and adult mental (sometimes physical) health issues may stem from what has happened but been unseen or intentionally kept hidden in kindergarten, with suspicions that during that period children were under duress and hence not capable of proper and adequate communication about abuse issues with their guardians in kindergarten.

## Developing kindergartener-specific questionnaire

In the Netherlands, where sex education is started in kindergarten, teachers guide children to properly name body parts and discuss various hypothetical scenarios to increase their body awareness. Children are guided to set their own limits that should never be crossed by anyone, and develop skills to protect against sexual coercion, intimidation and abuse. Moreover, children are asked about the kind of intimacy that occurs at home, so teachers could detect suspicious sexual misconduct. This approach, despite functioning well in many situations, could be further revised and improved into a universal instructional tool that uses an interactive approach, without needing much (ideally, any) ability of expression, to survey children about a decent list of items which may be associated with sexual or other misconduct. For example, parents can act as the teacher and encourage the kid to reconstruct what had happened in kindergarten, which should also be carried out in a reverse direction. This kind of instructional tools may be more suitable than other commonly used survey methods that only teachers and/or parents are interviewed and/or asked to fill out traditional questionnaires on behalf of the children who lack the capacity to express their will clearly. In particular, when we study certain topics such as child abuse, teachers or parents may bias or misreport facts intentionally or unintentionally if an effective tool is lacking. However, developing such a professional and interactive tool necessitates the involvement of pertinent experts, such as clinical psychologists, as well as the consideration of different forms of child abuse (e.g., neglect, physical, sexual, etc.) due to different epidemiology. While completing this kindergartener-specific questionnaire, children should be psychologically encouraged and guided to overcome falsehoods and/or intimidation, which often accompany the abuse.

## Traditional national offender registry systems

More importantly, this kindergartener-specific questionnaire should be popularized in a modern way at a large scale for catching those negative cases we intend to prevent from occurring in every kid's life. Furthermore, child abuse experiences would be more preventable if the risks identified could be timely mapped and made available and searchable to the public. Traditional questionnaire-based health survey tools and studies have been still implemented in a limited scope, even employing modern technologies (e.g., wearable sensors and smart phones), which can only ease the process of data collection to some extent. Geographic Information Systems (GIS) have significantly improved storage, integration, representation, analysis, interpretation, and dissemination of health data at large scales (8). Some GIS-based sex offender registration and notification systems have been developed to register crime in many countries over the past two decades, since the United States' first national-level sex offender registration law was passed in 1994 (9). For example, in the US Dru Sjodin National Sex Offender Public Website (NSOPW) which provides information to the public on the whereabouts of registered sex offenders across the country, entering an address, and selecting a distance to search for offenders by location can return all offenders with a registered address that is within the specified radius around the location searched. Nowadays, moreover, a NSOPW mobile app is available to provide users with information about registered sex offenders that have residential, employment, or school addresses that are within a close proximity (0.25-, 0.5-, or 1-mile) of the mobile device running the app. The US sex offender registration and notification now consists of individual registries and public registry websites operated by all 50 States, the District of Columbia, four of the principal US Territories, as well as over 70 federally-recognized Indian Tribes. These jurisdiction-level systems are linked in a national public registry website (available to the public) and also in a more detailed law enforcement database, both of which are administered through the US Department of Justice.

This kind of public registry system for general crime and especially child abuse in many countries, however, has not been well established or coordinated across the country as the US one. Also, there are two major weaknesses for the existing national registry systems, even for the mobile apps. One is that such system and operationalization is usually updated quarterly or even annually in most countries, so can only record crime reported in the past, with limited capacity to disclose timely information and risk and especially those under-investigated or intentionally hidden abuse experiences which are extremely important to the children population who lack the capacity to express their will clearly. The other weakness is that the search for potential risks are limited by country or, in most cases, state/provincial boundaries, given that offenders are not required to report their (international) travel notice in many countries. As globalization progresses and international traveling increases, for more effective preventive purposes, the crime information should be searchable to the global community instead of only to the local public. An additional shortcoming of the national registry system, assuming the importance of the kindergartener-specific questionnaire is recognized, is the difficulty in taking advantage of this survey tool due to functioning at different levels: traditional offender registry systems work at a national level only by authorities while the kindergartener-specific questionnaire work at a citizen level, overseen by authorities.

## What is suggested—utilizing citizen-based volunteered GIS technology

The Volunteered GIS (VGIS), also known as Crowd-sourcing, Collaborative or Participatory GIS, is the harnessing of modern technologies and tools to create, assemble, and disseminate geographic data provided voluntarily by any and every individual, such as WikiMapia, BikeMaps (10) and OpenStreetMap (11). A large number of smartphone apps have been developed to provide users with pertinent services and attractive features (e.g., finding paths and places of interest), as well as in health survey studies (e.g., measuring physical activity).

Child abuse prevention efforts, nevertheless, have comparatively less benefited from the VGIS technology. With the support of VGIS, every parent (or teacher) can have access to this interactive, kindergartener-specific questionnaire for monitoring how children have been educated and treated and detecting any kind of abuse occurring in kindergarten (or at home) in good time. One of the most important elements of VGIS in contrast to standard user-generated content is the geographic element. Therefore, any abuse case, once detected, can be reported with precise geographic location, so teachers/parents could be aware of surrounding dangers for prevention purposes. As globalization advances, some mobile populations in all occupations are increasingly becoming disproportionately vulnerable for these dangers due to lack of local information, such as exchanging and visiting scholars abroad in academia, company employees transferred to overseas offices, and rural parents leaving their children when moving to urban areas to seek work (so-called “left-behind children”). VGIS can create a global patchwork of child abuse information, and also help to track the progress of the cases and the movement of abusers, given that a large proportion of the crimes committed can be attributed to recidivists (12). For example, some disgraced teachers may resign before they could be dismissed, so it is possible for them to relocate in a new region or country where the background checking system is different or used sparingly, like one from Vancouver of Canada who was recently found to have worked in a prestigious school in Beijing of China since 2011. However, crowdsourcing technology could also create problems of intentional or unintentional misreporting on the “victim's” end. With the geographic element of reporting, spatial clustering of more than one report of misconduct may imply better validity of reporting.

## Key ethical issues

On the other hand, this could be a potential minefield if the confidentiality at any stage is inappropriately violated. The debate between pros and cons of public sex offender registries remains (13–20). A careful protocol must thus be discussed thoroughly among multiple stakeholders, including solving several key issues, such as: (1) suspects in suspicious incidents positioned and posted by citizen users should be anonymized and only available to certain groups (e.g., local polices) before any formal investigation; (2) citizen users posting suspicious incidents should be identifiable by certain groups (e.g., different levels of administrators responsible for target locations) for investigation purposes; (3) before confirmation, actual locations of suspicious incidents should be offset toward a random direction by a maximum of 2 kilometers in urban areas and a maximum of 5 kilometers for rural areas, for example; and (4) after confirmation, the information of criminals may be to different extents available to various groups (e.g., the full identity might be only available to local polices and kindergarten/school administrators). More issues need to be solve at later stages, such as how to operationalize and adapt this platform to local policies and regulations.

## Conclusion

Such a VGIS-based interactive survey tool will be an effective crime and mental health screening tool, and will help to make enormous strides in health data collection in the Big data Era. In addition, data collected will serve as a baseline for various life-course cohort studies, for example, environmental exposure, behavioral and non-communicable disease studies (21). Different sectors and stakeholders (e.g., educators, public health professionals, polices) in all countries can also easily coordinate and collaborate on pertinent problem-solving and policy-making strategies. More importantly, such tools integrating crowdsourcing technology with traditional practices of health data collection hold potential to be expanded to all types of crime-stopping and disease prevention worldwide, and gradually become a powerful tool of global health and epidemiological criminology (22). Citizens can be used as powerful sensors, if well managed.

## Author contributions

PJ confirms being the sole contributor of this work and approved it for publication.

### Conflict of interest statement

The author declares that the research was conducted in the absence of any commercial or financial relationships that could be construed as a potential conflict of interest.
